# High Recovery Chromatographic Purification of mRNA at Room Temperature and Neutral pH

**DOI:** 10.3390/ijms241814267

**Published:** 2023-09-19

**Authors:** Rok Miklavčič, Polona Megušar, Špela Meta Kodermac, Blaž Bakalar, Darko Dolenc, Rok Sekirnik, Aleš Štrancar, Urh Černigoj

**Affiliations:** 1 Sartorius BIA Separations d.o.o., Mirce 21, 5270 Ajdovščina, Slovenia; 2 Faculty of Medicine, University of Ljubljana, Vrazov trg 2, 1000 Ljubljana, Slovenia

**Keywords:** mRNA stability, nucleic acids separation, liquid chromatography, chromatographic monoliths, weak anion-exchanger, preparative chromatography, platform purification, isoelectric point

## Abstract

Messenger RNA (mRNA) is becoming an increasingly important therapeutic modality due to its potential for fast development and platform production. New emerging RNA modalities, such as circular RNA, drive the need for the development of non-affinity purification approaches. Recently, the highly efficient chromatographic purification of mRNA was demonstrated with multimodal monolithic chromatography media (CIM^®^ PrimaS), where efficient mRNA elution was achieved with an ascending pH gradient approach at pH 10.5. Here, we report that a newly developed chromatographic material enables the elution of mRNA at neutral pH and room temperature. This material demonstrates weak anion-exchanging properties and an isoelectric point of 5.3. It enables the baseline separation of mRNA (at least up to 10,000 nucleotides (nt) in size) from parental plasmid DNA (regardless of isoform composition) with both a NaCl gradient and ascending pH gradient approach, while mRNA elution is achieved in a pH range of 5–7. In addition, the basic structure of the novel material is a chromatographic monolith, enabling convection-assisted mass transfer of large RNA molecules to and from the active surface. This facilitates the elution of mRNA in 3–7 column volumes with more than 80% elution recovery and uncompromised integrity. This is demonstrated by the purification of a model mRNA (size 995 nt) from an in vitro transcription reaction mixture. The purified mRNA is stable for at least 34 days, stored in purified H_2_O at room temperature.

## 1. Introduction

Messenger RNA (mRNA) has distinguished itself as a prominent therapeutic modality with enormous potential in the fields of vaccination, cancer therapy, and personalized medicines [1,2], as demonstrated by vaccines against SARS-CoV-2 [3]. Furthermore, new RNA modalities, such as self-amplifying RNA (saRNA) and circular RNA, are emerging with a potential to further progress RNA therapeutics towards higher stability in formulation and in vivo [4]. Drug substance purity underpins a safe and effective use of mRNA technology; therefore, robust and efficient purification solutions must be implemented to remove process-related impurities, such as components of in vitro transcription (IVT) reaction [5], or host-derived biomolecules in the case of in vivo bioproduction of mRNA, which has been postulated as a potential alternative to IVT [6].

The purification process must additionally ensure drug substance stability, homogeneity, and integrity. The selection of appropriate purification conditions is especially crucial for mRNA, as it is very susceptible to hydrolysis of phosphodiester bonds compared to DNA, due to the presence of 2′-OH group in ribose [7,8,9,10]. Alkaline pH, elevated temperatures, and metal ions are the main environmental factors jeopardizing mRNA integrity [7,8,11,12], with depurination, oxidation, and deamination of mRNA being common degradation mechanisms that may impact its therapeutic application [8,13]. For an effective mRNA purification process, high temperatures and prolonged exposure to extreme pH should therefore be avoided.

The industrial purification of biomolecules typically employs at least one chromatographic step, intended for the selective, efficient, and scalable separation of target molecules from impurities. Chromatographic monoliths and membranes have demonstrated superior properties compared to particle-based materials for the purification of large therapeutic biomolecules, such as plasmid DNA (pDNA) and mRNA, without compromising their integrity [14,15]. Due to large channel size and convection-based analyte mass transport to and from the stationary phase surface, these materials allow flow-independent performance, high sample recovery, and fast processing times, while maintaining low shear stress [16]. mRNA purification using chromatographic monoliths ensures a stable mRNA drug substance [17,18,19], improving mRNA stability compared to standard precipitation approaches [15].

Various chromatographic approaches have already been implemented for single-stranded RNA (ssRNA) purification, but each one with clear limitations [20]. The main drawbacks of currently applied methods are the following: low elution recoveries without applying harsh purification conditions, such as high elution temperature [21] or high pH (anion-exchange chromatography) [22]; large concentrations of salts in buffers (hydrophobic interaction chromatography) [23]; organic solvents in buffers (reverse-phase chromatography) [24,25]; and sequence specificity (affinity chromatography) [18,26].

According to our literature search, the multimodal weak anion-exchanging monolith (CIM^®^ PrimaS) is the only existing chromatographic anion exchanger with demonstrated separation of various nucleic acids (including pDNA, double-stranded RNA, transfer RNA, mRNA) and the platform purification of milligrams of mRNA up to at least 10,000 nucleotides (nt) at room temperature, but high elution recovery is only achieved at a pH of between 10 and 11 [17,19]. The elution mechanism is based on switching off the positive charge of the weak anion exchanger by increasing the buffer pH sufficiently above the ligand’s acid dissociation constant (pKa) value [27]. The relatively high elution pH could be a potential drawback when purifying constructs susceptible to hydrolysis [7,12]. Opting for anion-exchanging materials, which lose their positive charge around neutral pH range, should consequently provide the possibility of eluting nucleic acids under native conditions. This was previously shown with chitosan, where the binding of DNA and RNA was achieved at pH 5.0 and elution at pH 9.0 [28,29,30,31]. This material was successfully implemented for the analytical extraction of nanograms of DNA [30] and RNA [31], but is not appropriate for scalable liquid chromatography use. Similarly, solid phases functionalized with various amphoteric ligands, e.g., Bis-Tris, polyhistidine, and tricine, enabled the purification of DNA by applying biological samples at acidic pH and eluting DNA at pH 8.5 [32]. However, the described ligands have not yet been specifically evaluated for mRNA purification.

Zeta potential analysis is a practical technique for studying the surface properties of materials and can provide significant insight about their surface charge under various conditions. A material’s isoelectric point (IEP) represents the pH at which the material has a net charge of zero [33] and is therefore connected with surface protonation and deprotonation processes. IEP could be crucial for selecting chromatographic materials, which would enable mRNA purification under mild elution conditions—neutral pH and room temperature [34,35].

The aim of the present investigation was, therefore, to develop an efficient and easily scalable mRNA purification approach based on a carefully selected chromatographic monolith possessing a chemically stable ligand with weak anion exchanging properties with an IEP between 5 and 8, which should result in a stable mRNA with high integrity.

## 2. Results

### 2.1. Zeta Potential Analysis of Monoliths

**Figure 1:**
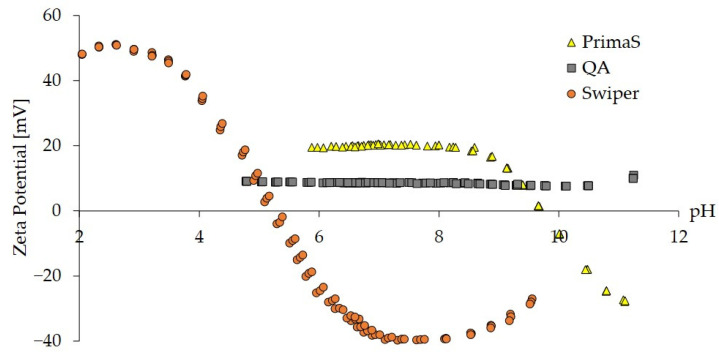
Zeta potential of chromatographic monoliths as a function of pH.

The aim of the initial experiment was to apply zeta potential analysis to select a cationic monolith material with an IEP between pH 5 and 8. Zeta potential as a function of pH in the range of 2–11 was determined for three monolith materials, differing in the type of surface-conjugated ligands (Figure 1). CIM QA, a strong anion exchanger, demonstrated a constantly positive zeta potential of around +8.5 mV (Figure 1, gray squares), while CIM PrimaS behaved as a weak anion exchanger. Its surface positive charge decreased above pH 8.5 with an IEP around 9.7 (Figure 1, yellow triangles). CIM Swiper, a new prototype chromatographic monolith, on the other hand, displayed an IEP of 5.3 and an increasingly positive net charge below pH 5.3 (Figure 1, orange circles). The isoelectric point of CIM Swiper was approximately 4.5 units lower compared to CIM PrimaS, indicating its potential applicability to elute mRNA at neutral pH.

### 2.2. Chromatographic Evaluation of CIM Swiper for mRNA Separation

**Figure 2:**
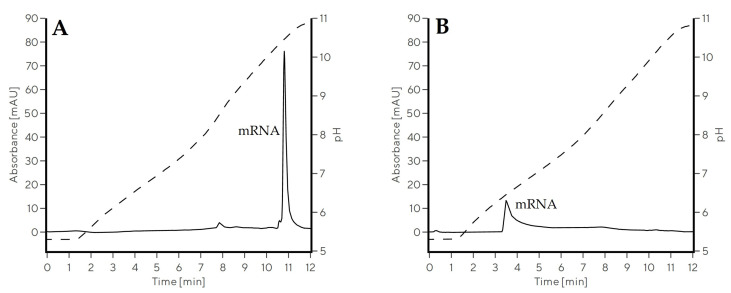
Elution profile of 1 µg of eGFP mRNA from 0.1 mL CIMmic PrimaS disk (**A**) and 0.1 mL CIMmic Swiper disk (**B**) using Gradient 1. Solid line represents the absorbance measured at 260 nm, while dashed line represents pH trace.

CIM Swiper was evaluated for the elution of mRNA in ascending pH gradient and benchmarked against CIM PrimaS. mRNA encoding green-fluorescent protein (eGFP mRNA, size 995 nt) was used as a model mRNA and evaluation was performed on 0.1 mL screening CIMmic disks. A broad linear pH gradient from 5.0 to 11.0 over 100 column volumes (CVs) (Gradient 1) using a four-component MES/HEPES/BTP/CAPS buffer system at low conductivity was applied to cover the broad span of IEP values of both materials in a single method. The pH at mRNA elution was approximately 6.6 with CIM Swiper (Figure 2B), nearly 4 pH units lower than with CIM PrimaS (Figure 2A), while the difference in elution pH correlated with the IEP difference of the two materials. mRNA eluted approximately 1 pH unit above their isoelectric points with both materials. This demonstrated a potential functionality of a chromatographic material with a low IEP (such as CIM Swiper) for mRNA purification at neutral pH and room temperature.

The elution peak of mRNA from CIM Swiper was approximately 2.5 times broader compared to PrimaS during the preliminary evaluation of the material (Figure 2), indicating inefficient elution conditions.

### 2.3. Optimization of mRNA Elution and mRNA–pDNA Separation

Ineffective elution required an optimization of the chromatographic performance of the new material. In parallel, the evaluation and optimization of conditions for the separation of mRNA from pDNA (mRNA–pDNA separation) was performed, because pDNA is used in IVT reaction as a template for T7 polymerase-catalyzed transcription to RNA and is therefore a process-derived impurity [1,2]. eGFP mRNA and pUCBS4.7 pDNA (size 4.7 kbp) were used as representative analytes.

#### 2.3.1. Optimization of the pH Gradient Approach

Various salts were screened as additives to ascending pH gradient as this was previously shown to influence the separation of nucleic acids [17,23]. The buffer composition was fixed at 10 mM MES, 10 mM HEPES to enable a linear pH gradient in the range of 5.5–8.0, where mRNA elution was expected. Salts (NaCl, Na-phosphate, Na-citrate, and Na-EDTA) were added to both binding and elution buffers at 50 mM concentration to identify additives with considerable impact on separation (Table 1, Figure S1).

The control experiment with no additives (Gradient 2) showed poor separation of mRNA and pDNA; resolution factor (*R*) was 0.4 (Table 1) with a broad elution volume for both components. Nonetheless, pDNA was retained less than mRNA, eluting at a pH approximately 0.14 units lower than mRNA. NaCl (Gradient 3) significantly decreased the elution volume and improved the separation of both components. Na-phosphate (Gradient 4), and especially Na-citrate (Gradient 5) and Na-EDTA (Gradient 6), drastically improved the separation of pDNA and mRNA, achieving a resolution factor above 5 while simultaneously decreasing the elution pH of both nucleic acids, compared to the control experiment (Table 1, Figure S1).

**Figure 3:**
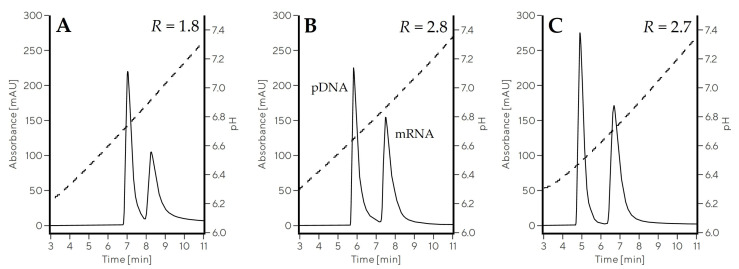
Elution profiles showing the effect of anions on mRNA–pDNA separation using CIMmultus Swiper 1 mL column in pH gradient from 6.0 to 7.5. Gradient 7 (**A**) and Gradient 8 (**B**) were performed at approximately 6–7 mS/cm, while conductivity in Gradient 9 (**C**) was around 16 mS/cm. Solid line represents the absorbance measured at 260 nm, while dashed line represents pH trace.

The addition of all four additives (NaCl, Na-phosphate, Na-citrate, Na-EDTA) affected the increase in the buffer conductivity, which could hypothetically be the only reason for improved separation. To demonstrate that specific anions, and not just conductivity, influenced mRNA–pDNA separation, we compared this separation in a fixed pH range using different buffer systems, but keeping conductivity the same. Buffer composed of 10 mM MES, 10 mM HEPES, and 50 mM NaCl (Gradient 7) was compared to 20 mM Na-citrate, 20 mM Na-phosphate buffers (Gradient 8) in the same pH range at a conductivity of approximately 6–7 mS/cm, but this time using CIMmultus Swiper columns with 1 mL bed volume (Table 2, Figure 3). The mRNA–pDNA separation was substantially improved in Gradient 8 (*R* = 2.8, Figure 3B) compared to Gradient 7 (*R* = 1.8, Figure 3A), indicating an additional contribution of phosphate and citrate to pDNA–mRNA separation beyond the effect of a higher conductivity. Only when 150 mM NaCl was supplemented to the MES/HEPES buffer system (Gradient 9) and separation performed at 16 mS/cm was a similar separation efficiency to Gradient 8 achieved (*R* = 2.7, Figure 3C).

#### 2.3.2. Evaluation of NaCl Gradient for Nucleic Acid Elution

Standard approaches for nucleic acid purification and separation using anion exchangers employ salt gradients for elution, e.g., NaCl gradient for pDNA purification using chromatographic materials such as CIM DEAE, CIM QA, Mustang Q, and Sartobind Q [17,36,37]. As this is an established elution approach, we performed mRNA–pDNA separation with CIM Swiper using an ascending NaCl gradient at pH 6.0 (Gradient 10) and pH 5.0 (Gradient 11). Stronger binding strength was expected at pH 5.0 based on the zeta potential curve of CIM Swiper (Figure 1).

**Figure 4:**
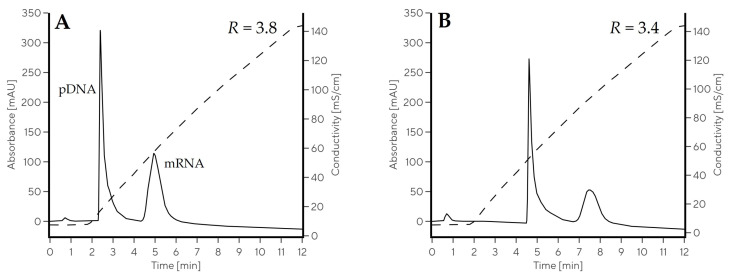
Comparison of mRNA–pDNA separation in NaCl gradients at pH 6.0 ((**A**), Gradient 10) and pH 5.0 ((**B**), Gradient 11) using CIMmultus Swiper 1 mL column. Solid line represents the absorbance measured at 260 nm, while dashed line represents conductivity trace.

The retention of both nucleic acids by CIM Swiper was indeed enhanced at pH 5.0, proven by approximately 0.5 M higher eluting NaCl concentration regardless the type of nucleic acid. The baseline separation between pDNA and mRNA was achieved in NaCl gradient at both evaluated conditions (Figure 4). mRNA eluted at an approximately 0.6 M higher NaCl concentration than pDNA regardless the pH. The resolution factor between both components was above 3.4 in NaCl gradients, approximately 1 unit higher than in comparable pH gradients (Table 2). However, the mRNA eluted in a narrower peak in pH gradients (w_0.5_ < 0.5 min) compared to NaCl gradients (w_0.5_ > 0.6 min) and the sharpest mRNA elution peak was achieved in Gradient 8, when both phosphate and citrate were present in the buffers. This was the reason to keep the pH elution approach for mRNA in the following experiments (with both citrate and phosphate present), enabling its higher concentration factor. On the other hand, the NaCl gradient was selected for double-stranded DNA (dsDNA) clearance due to a more robust and selective separation from mRNA. Combined salt- and pH gradient approaches were implemented in the mRNA purification experiments to demonstrate the potential of CIM Swiper for separating and purifying mRNA from IVT process samples.

#### 2.3.3. Separation of ssRNAs up to 10,000 nt in Size

**Figure 5:**
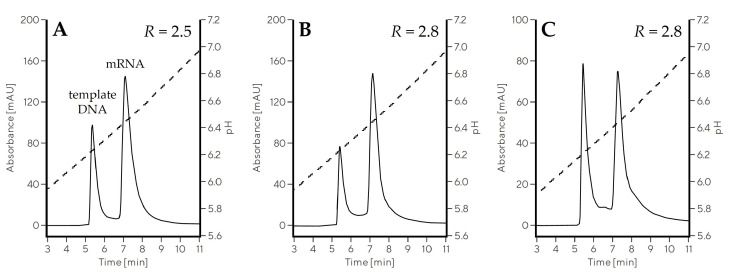
Separation of RNAs of different sizes from their respective linear DNA templates using Gradient 8 for eGFP mRNA ((**A**), size 995 nt), mFIX mRNA ((**B**), size 4000 nt) and saRNA ((**C**), size 10,000 nt). Solid line represents the absorbance measured at 260 nm, while dashed line represents pH trace.

The CIM Swiper prototype was lastly evaluated for the separation of different sizes of ssRNA molecules, to prove its constant chromatographic efficiency and recovery regardless RNA size. Three in vitro transcribed ssRNAs (two mRNAs with the size of 995 nt and 4000 nt and one saRNA with the size of 10,000 nt) were separated from their respective linear DNA templates (size 3.3–12 kbp) using the optimized conditions from Gradient 8 (Table 3, Figure 5). All molecules eluted at a similar pH with the ssRNA-DNA resolution between 2.5 and 2.8 regardless the sample analyzed. Furthermore, the elution recovery of ssRNA was above 80% for all three constructs, demonstrating efficient and almost size-independent elution.

### 2.4. Chromatographic Purification of mRNA from IVT Reaction Mixture at Room Temperature

**Figure 6:**
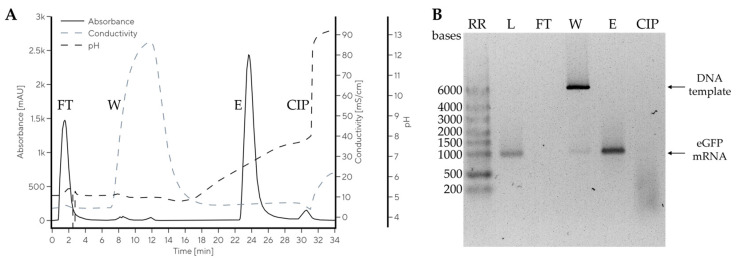
mRNA purification from IVT reaction mixture using CIMmultus Swiper 1 mL column. Preparative chromatogram (**A**) and AGE analysis of the collected fractions (**B**). RR—RiboRuler High Range RNA Ladder (Thermo Fisher Scientific, Waltham, MA, USA), L—load, FT—flow-through fraction, W—salt wash fraction, E—elution fraction, CIP—CIP fraction. AGE loading mass was 80 ng.

Separation of mRNA and pDNA was next extended to purification of mRNA from IVT reaction mixture, containing impurities such as nucleoside triphosphates (NTPs), enzymes, and DNA template. The downstream processing step was designed to bind both DNA template and target mRNA (1.5 mg) to CIM Swiper (1 mL column) at pH 5.0, and then selectively eliminate the DNA template from the column with a 1 M NaCl wash at pH 5.0 and, finally, elute pure mRNA by increasing pH to 7.5 (Figure 6A).

The determined dynamic binding capacity of CIM Swiper for mRNA from IVT reaction was approximately 2–3 mg of mRNA per mL of monolith (results not shown); therefore, the preparative run was performed at approximately 50–60% of column saturation. The IVT sample was applied to the column in 20 mM Na-citrate, 20 mM Na-phosphate, pH 5.0, where only DNA template and eGFP mRNA bound to the column. No mRNA or DNA were detected in the flow-through (FT) fraction with agarose gel electrophoresis (AGE, Figure 6B) or chromatographic CIMac PrimaS analytics (Figure S2), demonstrating no overloading of the column. The NTPs did not bind to the column and were only detected in the FT fraction (Figure S2). A wash with buffer containing 1 M NaCl (W) at pH 5.0 was applied to remove the DNA template, but also eluted trace amounts of eGFP mRNA (Figure 6, W). Impurity-free mRNA was eluted by raising the buffer pH up to 7.5, while mRNA elution pH was approximately 6.6, in line with the model system (Table 1). mRNA elution recovery of 93% was determined in the main elution fraction by CIMac PrimaS analytics. The elution volume was 7.3 CVs with an mRNA concentration of 192 µg/mL. This experiment demonstrated an efficient purification of mRNA with CIM Swiper at room temperature, without exposing the mRNA to basic pH.

### 2.5. mRNA Stability Study

A larger amount of mRNA was needed for a stability study, and this was an excellent opportunity to probe the possibility of scaling up the developed mRNA purification process at the same time. Therefore an 800 mL CIMmultus Swiper column was loaded with an IVT mixture containing 664 mg of eGFP mRNA. During purification, 547 mg of eGFP mRNA (83% of the loaded amount) was successfully recovered from the column in a pH elution step into a pH 7.5 buffer with a slightly modified preparative method, as described in the Section 4. The concentration of eGFP mRNA in the main elution fraction was 195 µg/mL, corresponding to an elution volume of 3.5 CVs.

**Figure 7:**
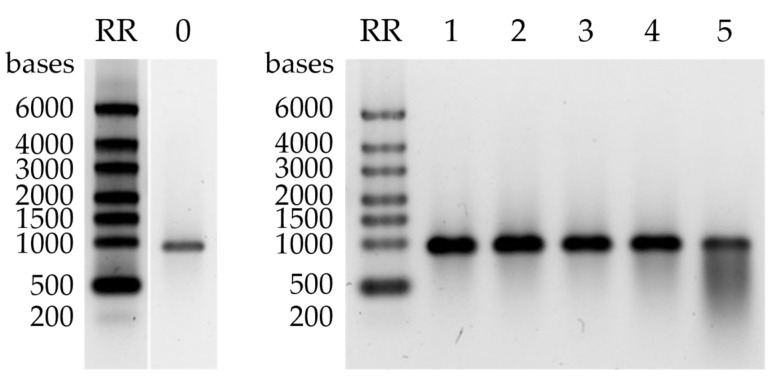
Stability study of eGFP mRNA purified with CIMmultus Swiper 800 mL column. AGE of the eGFP mRNA sample after purification (lane 0) and after storage at −80 °C (lane 1), −20 °C (lane 2), 4 °C (lane 3), room temperature (lane 4), and 37 °C (lane 5) for 34 days. RR—RiboRuler High Range RNA Ladder (Thermo Fisher Scientific).

The purified eGFP mRNA was buffer exchanged into purified H_2_O and subjected to a stability study to determine its integrity after purification and storage. When the mRNA was stored at room temperature or below (4 °C, −20 °C and −80 °C) for 34 days, no mRNA degradation was observed by AGE, while exposure to 37 °C led to its partial degradation (Figure 7).

## 3. Discussion

mRNA’s purity and integrity pave the way for its safe and efficient therapeutic application, while chromatography is a relatively efficient tool to remove process- or product-related impurities in a scalable manner [15,18,19,24]. Currently, the most advanced chromatographic way of exploiting weak anion-exchanging stationary phases for mRNA elution involves the decharging of CIM PrimaS columns in ascending pH gradient [19]. Due to the high IEP (9.7, Figure 1) of the material, the PrimaS-based chromatography requires pH 10.5 to efficiently elute mRNA [19], which may exacerbate the inherent instability of longer RNA constructs.

Here, we report that decreasing the IEP of a weak anion-exchanging chromatographic monolith positively influences the pH of mRNA elution. A new chromatographic monolith prototype, CIM Swiper, demonstrates weak anion-exchanging properties and an IEP of 5.3; therefore, the loss of its positive surface charge already takes place at slightly acidic pH, enabling the desorption of negatively charged mRNA below pH 7 with pH gradient elution approach (Figure 2). The difference in the elution pH of mRNA between CIM Swiper and PrimaS correlates with their observed difference in IEP. Additionally, mRNA is eluted at a pH approximately 1 unit above their IEP with both materials, possibly indicating that they interact with mRNA in a similar manner. Furthermore, our results demonstrate the importance of zeta potential analysis as a proxy for the estimation of the elution behavior of ion-exchange chromatographic materials for specific applications.

Next, we optimized the elution of mRNA and the separation of mRNA from its main process-related impurity, dsDNA. An ascending pH gradient alone, with no ions present, results in the poor separation of mRNA and pDNA with a broad mRNA elution peak. The idea behind implementing additional salts in working buffers was to shield the electrostatic interaction between nucleic acids and the chromatographic material as well as to promote the stabilization of the RNA structure, as variation in both parameters could be the reason for the observed broadening of the peak. NaCl was selected as the simplest ionic salt, broadly used as an eluent in ion-exchange chromatography, which also stabilizes the secondary and tertiary structure of RNA [38]. The addition of NaCl improves mRNA–pDNA separation, reduces mRNA elution volume, and decreases mRNA elution pH. It was previously shown that phosphate salts are more efficient than chloride salts at eluting proteins and nucleic acids from CIM PrimaS [19], and our observations imply that this could also be the case for CIM Swiper. We demonstrate, that the addition of low concentrations of common multivalent anions Na-phosphate, Na-citrate, and Na-EDTA to pH gradient buffers improve the separation of mRNA from DNA and simultaneously reduce mRNA elution volume. The addition of all four additives (NaCl, Na-phosphate, Na-citrate, Na-EDTA) affects the increase in buffer conductivity differently, but the improvement in chromatographic performance is undoubtedly related to both the conductivity increase and chemical nature of the anions themselves. When compared at the same buffer conductivity, a buffer combination of Na-citrate and Na-phosphate enables better separation of mRNA and pDNA compared to a buffer combination of MES and HEPES with the addition of NaCl, while increasing the salt molarity further improves the separation. The influence of anions on nucleic acid chromatographic separation using CIM Swiper could be an important future study from a mechanistic point of view. We presume their effect stems mainly from their interaction with the chromatographic surface and could be a consequence of their low charge density and large hydration shell compared to chloride.

mRNA–pDNA separation can also be achieved with standard ascending NaCl gradients at a fixed pH, while pH in the range of 5–6 profoundly influences the retention of both nucleic acids. This behavior is expected and can be explained by the zeta potential curve (Figure 1), as the surface charge of CIM Swiper increases at lower pH, resulting in stronger electrostatic interaction with nucleic acids. The pH variation in this range also influences the binding capacity of CIM Swiper for nucleic acids, which is higher at more acidic pH. For example, the dynamic binding capacities at 50% breakthrough for the model pUCBS4.7 plasmid were 1.2 mg of plasmid per mL of monolith and 1.7 mg of plasmid per mL of monolith at pH 6 and 5, respectively, demonstrating a 40% increase in the binding capacity if pH is decreased from 6 to 5. Several subsequent runs and CIP steps were performed on the same column with no observed influence on chromatographic performance, indicating that the ligand is sufficiently stable in 1 M NaOH for at least 6 h.

An optimized approach for the purification of mRNA from an IVT reaction mixture using CIM Swiper was built on acquired knowledge and was confirmed by purifying approximately 1.5 mg of IVT eGFP mRNA on a 1 mL chromatographic column at a loading pH of 5. NTPs do not bind to the material at this pH, which is an additional benefit facilitating the removal of these process-derived impurities (Figure S2). After binding, a 1 M NaCl wash at pH 5 is utilized for DNA template removal, as the selectivity of mRNA and pDNA separation is improved with a salt gradient. The experiment demonstrates trace amounts of eGFP mRNA in the wash fraction, but this loss could be avoided by optimizing the wash conditions by decreasing the NaCl concentration in the buffer. After removing all impurities, eGFP mRNA is eluted at pH 6.6 by an ascending pH gradient, achieving a 93% elution recovery. pH gradient elution is preferred for mRNA elution due to a lower mRNA elution volume compared to NaCl gradient elution. This is probably due to the complete and efficient elimination of electrostatic interactions between mRNA and chromatographic surface by the pH gradient, while shielding of electrostatic interactions with a salt gradient at a fixed pH is less efficient. In older literature, pH levels of 8.5 [32] or 9.0 [30,31] were required for the elution of DNA from stationary phases containing ligands with low pKa values, such as chitosan, Bis-Tris, polyhistidine, and tricine. Here, we demonstrate that DNA and mRNA can be eluted even below pH 7 if a material with sufficiently low IEP is utilized. Additionally, the clear potential of the monolith-based stationary phase to efficiently purify mRNA in milligram amounts below pH 7 was shown. The process could be scaled up to be applicable for industrial mRNA production, which was previously not attainable using materials containing low pKa ligands. The scalability of our purification approach was already demonstrated by successfully purifying 547 mg of eGFP mRNA with an 800 mL CIMmultus Swiper monolithic column. We estimate that such column size could already support the purification of up to 2.4 g of mRNA, sufficient for approximately 24,000 doses of mRNA-1273 [39] or 80,000 doses of BNT162b2 [40] mRNA vaccines against SARS-CoV-2 in a single chromatographic run.

RNA size could impact its purification efficiency, as evident when comparing the elution of 10,000 nt saRNA to eGFP mRNA (995 nt), the former having approximately 15% lower elution recovery. However, the elution pH and separation from DNA were not influenced by ssRNA size, which clearly demonstrates the possibility for separating and purifying large RNAs as well as large DNAs using the developed approach. According to our literature review, this is the first demonstration of an efficient chromatographic separation of saRNA. The optimization of chromatographic conditions or even the material itself could be evaluated in the future for improved performance with large constructs.

CIM Swiper enables the purification of similar amounts of mRNA compared to affinity chromatography due to similar dynamic binding capacity [26], but additionally enables the purification of non-polyadenylated RNA constructs (results not shown) due to the utilized anion-exchange purification principle. With plasmids, elution conditions are not influenced by their structure, as linear and circular forms of the same plasmid elute at similar retention times. This could indicate potential for the purification of circular RNA from nucleotides and DNA template with the developed approach, as a circular structure is not expected to greatly influence its interaction with anion-exchange materials [20].

During purification with the developed procedure, mRNA is constantly subjected to room temperature and a mildly acidic to neutral pH range (5.0–7.5), which is the optimal pH range stability-wise [41]. The stability study confirms that the integrity of the purified mRNA is unaltered and the purification process does not affect its stability, similar to mRNA from affinity [15] and multimodal chromatography purification [19]. The 34-day room temperature stability of CIM Swiper-purified mRNA demonstrates that this purification approach does indeed promote high mRNA integrity, which is beneficial for therapeutic RNA.

## 4. Materials and Methods

### 4.1. Chemicals and Reagents

The buffers were freshly prepared with European Pharmacopoeia grade purified H_2_O and analytical grade reagents. The buffer solutions were filtered through a 0.2 μm PES filter (Nalgene Rapid-Flow, Thermo Fisher Scientific).

Sodium chloride (NaCl) and sodium hydroxide (NaOH) were purchased from Honeywell (Charlotte, NC, USA). Citric acid monohydrate and disodium salt of ethylenediaminetetraacetic acid (EDTA) were purchased from Merck (Darmstadt, Germany). Hydrochloric acid (HCl), tris(hydroxymethyl)aminomethane (TRIS), sodium phosphate monobasic dihydrate (NaH_2_PO_4_·2H_2_O), 3-(Cyclohexylamino)-1-propanesulfonic acid (CAPS), 4-(2-hydroxyethyl)piperazine-1-ethanesulfonic acid (HEPES), Bis-TRIS-propane (BTP), sodium pyrophosphate tetrabasic decahydrate (Na_4_P_4_O_7_·10H_2_O), and agarose were purchased from Sigma Aldrich (St. Louis, MO, USA). Finally, 2-(N-morpholino)ethanesulfonic acid (MES) monohydrate was purchased from Carl Roth (Karlsruhe, Germany) and 96%(*v*/*v*) ethanol (EtOH) from Pharmachem (Ljubljana, Slovenia).

### 4.2. Chromatograhic Columns

CIMmic^TM^ Swiper, PrimaS, and QA disks (all from Sartorius BIA Separations, Ajdovščina, Slovenia) with 0.1 mL bed volume and 2 µm channel diameter were used for initial chromatographic screening. A CIMmultus^TM^ Swiper 1 mL monolithic column (Sartorius BIA Separations) was used for the optimization of mRNA–pDNA separation, while CIMmultus Swiper 1 mL and 800 mL columns were used for mRNA purification from the IVT reaction mixture. A CIMac^TM^ PrimaS-0.1 Analytical Column (2 µm) (Sartorius BIA Separations) was used for mRNA analytics.

### 4.3. Zeta Potential Analysis of Monoliths

Zeta potential measurements were performed with SurPASS 3 electrokinetic analyzer (Anton Paar GmbH, Graz, Austria) using the cylindrical cell. For zeta potential analysis, approximately 1 g of monolith was washed with 10 mL of 96% EtOH for 15 min and the EtOH was discarded. The monoliths were heated for 6 h at 60 °C until completely dry and then crushed coarsely by pestle and mortar. Then, 0.25 g of the crushed monolith was fixed in the cylindrical cell with the support disks and filters. The permeability index of the sample was adjusted to around 100 by rotating the micrometer screw. Here, 1 mM KCl solution was used as the electrolyte, while 0.05 M NaOH and 0.05 M HCl were used for adjusting the pH. The pH dependence of the zeta potential was determined in the pH range from 11 to 5 (CIM QA and CIM PrimaS) or from pH 10 to 2 (CIM Swiper) with a step of 0.3, with three rinse cycles and three zeta potential measurements at each pH value. A pressure gradient of 1500–1000 mbar was applied to generate streaming potential. SurPASS 3 software (Anton Paar GmbH) was used for system operation and data evaluation, with the zeta potential calculated via the established Helmholtz–Smoluchowski equation [41].

### 4.4. Preparation of Purified Nucleic Acids

pUCBS4.7 plasmid (size 4.7 kbp) and plasmids encoding eGFP (provided by BioMay AG, Vienna, Austria), mFIX mRNA (Sartorius BIA Separations), and saRNA (provided by TRON, Mainz, Germany), were isolated from *E. coli* paste by alkaline lysis and subsequent chromatographic purification employing an anion-exchange capture step with CIMmultus DEAE (Sartorius BIA Separations), as described elsewhere [37]. Plasmids were linearized by appropriate restriction enzymes (*Not*I-HF, *Bbs*I-HF, and *Sap*I for plasmids encoding eGFP, mFIX, or saRNA, respectively) following the manufacturer’s procedure (all enzymes were from New England Biolabs, Ipswich, MA, USA), purified with a CIMmultus C4 HLD column (Sartorius BIA Separations) by applying a descending ammonium sulphate gradient (2.5–0 M) in 50 mM Tris–HCl, 10 mM EDTA, pH 7.2, and finally buffer-exchanged into purified H_2_O (30 kDa Amicon MWCO filter, Merck). ssRNAs were synthesized from linear plasmids using a previously described IVT procedure in a thermal shaker and the synthesis process was quenched by the addition of EDTA [5,15]. To obtain pure mRNA samples for the screening of chromatographic conditions, polyadenylated mRNAs were purified by affinity chromatography using CIMmultus Oligo dT monolithic columns (Sartorius BIA Separations) as described before [5,18]. An EDTA-inactivated IVT reaction mixture was used as the initial sample for mRNA purification.

### 4.5. Chromatography

Chromatographic experiments were performed on a PATfix HPLC system (Sartorius BIA Separations) or ÄKTA pure 150 M (Cytiva, Marlborough, MA, USA) composed of binary or quaternary pumps, a multiwavelength UV–Vis detector (10 mm flow cell path length), a conductometer, and a pH monitor. UV absorbance was monitored at 260 nm and 280 nm. ClarityChrom (Knauer, Berlin, Germany) and UNICORN (Cytiva) software were used for instrument control and data acquisition, while PATfix software (Sartorius BIA Separations) was used for data analysis.

#### 4.5.1. Evaluation of CIMmic Disks in Ascending pH Gradients

Initial chromatographic experiments were performed on CIMmic disks—monolithic screening disks with 0.1 mL bed volume. The disks were stored in 20% (*v*/*v*) EtOH. Before testing, the disks were packed into CIMmic housing (Sartorius BIA Separations), flushed with 10 CV of purified H_2_O to eliminate storage solution, and then regenerated with 20 CV of 1 M NaOH. The column was then washed with 30 CV of 100 mM acetic acid, 1 M NaCl, pH 5.0, followed by 15 min incubation to enable column equilibration at pH 5.0. The columns were then washed with 10 CV of purified H_2_O, 30 CV of the appropriate buffer A, and mounted to the PATfix HPLC system for evaluation. Chromatographic runs were performed at a flowrate of 1 mL/min (10 CV/min) at room temperature. In general, methods comprised the application of the sample in buffer A, a wash with buffer A, followed by elution with a linear gradient from 100% buffer A to 100% buffer B, a hold at 100% buffer B, and column equilibration back to 100% buffer A.

Blank runs were performed by injecting MPA before and in between injections of samples. eGFP mRNA (1 µg loading amount) or pUCBS4.7 pDNA (2 µg loading amount) was separated on the column. The elution gradient slope, buffer composition, and buffer pH were varied during the evaluation, as described below.

Following gradients were used:

Gradient 1. Buffer A: 10 mM MES, 10 mM HEPES, 10 mM BTP, 10 mM CAPS, pH 5.5. Buffer B: 10 mM MES, 10 mM HEPES, 10 mM BTP, 10 mM CAPS, pH 11.0. CIMmic PrimaS (2 µm), and CIMmic Swiper (2 µm), linear gradient from 100% buffer A to 100% buffer B over 100 CV at a flowrate of 1 mL/min.

Gradient 2. Buffer A: 10 mM MES, 10 mM HEPES, pH 5.5. Buffer B: 10 mM MES, 10 mM HEPES, pH 8.0. CIMmic Swiper (2 µm), linear gradient from 100% buffer A to 100% buffer B over 50 CV at a flowrate of 1 mL/min.

Gradient 3. Buffer A: 10 mM MES, 10 mM HEPES, 50 mM NaCl, pH 5.5. Buffer B: 10 mM MES, 10 mM HEPES, 50 mM NaCl, pH 7.5. CIMmic Swiper (2 µm), linear gradient from 100% buffer A to 100% buffer B over 100 CV at a flowrate of 1 mL/min.

Gradient 4. Buffer A: 10 mM MES, 10 mM HEPES, 50 mM Na-phosphate, pH 5.5. Buffer B: 10 mM MES, 10 mM HEPES, 50 mM Na-phosphate, pH 8.0. CIMmic Swiper (2 µm), linear gradient from 100% buffer A to 100% buffer B over 50 CV at a flowrate of 1 mL/min.

Gradient 5. Buffer A: 10 mM MES, 10 mM HEPES, 50 mM Na-citrate, pH 5.5. Buffer B: 10 mM MES, 10 mM HEPES, 50 mM Na-citrate, pH 8.0. CIMmic Swiper (2 µm), linear gradient from 100% buffer A to 100% buffer B over 100 CV at a flowrate of 1 mL/min.

Gradient 6. Buffer A: 10 mM MES, 10 mM HEPES, 50 mM Na-EDTA, pH 5.5. Buffer B: 10 mM MES, 10 mM HEPES, 50 mM Na-EDTA, pH 8.0. CIMmic Swiper (2 µm), linear gradient from 100% buffer A to 100% buffer B over 100 CV at a flowrate of 1 mL/min.

Where the separation of mRNA and pDNA was of interest, the elution times of the main peak and the peak width at half height were collected after integration. The resolution between mRNA and pDNA (*R*) was calculated according to the European Pharmacopoeia, using Equation (1). Additionally, the elution pH of pDNA and mRNA were estimated by normalizing the observed elution pH to the chromatographic gradient and nominal pH values of buffers A and B using Equation (2). The offset between UV and pH detectors was heeded when obtaining the elution pH from the chromatograms.

(1) $$R_{m/\mathrm{ssRNA}-(p)\mathrm{DNA}}=1.18\times \frac{t_{m/\mathrm{ssRNA}}-t_{(p)\mathrm{DNA}}}{w_{0.5\;m/\mathrm{ssRNA}}+w_{0.5\;(p)\mathrm{DNA}}}$$

(2) $${pH(NA)}_{norm.}=\frac{{pH(NA)}_{exp.}-{pH(A)}_{exp.}}{{pH(B)}_{exp.}-{pH(A)}_{exp.}}\times \left(pH\left(B\right)-pH\left(A\right)\right)+pH\left(A\right)$$

In the equations, *R* is the chromatographic resolution factor, *t*_m/ssRNA_ and *t*_(p)DNA_ are the retention times of m/ssRNA and (p)DNA, and *w*_0.5 m/ssRNA_ and *w*_0.5 (p)DNA_ are the width at the half height of m/ssRNA and (p)DNA. pH(NA)_norm._ is the normalized elution pH of nucleic acids, pH(NA)_exp._, pH(A)_exp._, and pH(B)_exp._ are the pH values obtained from the chromatograms at the nucleic acid elution peak center, 100% buffer A, or 100% buffer B, respectively. pH(A) and pH(B) are the nominal pH values of buffer A and buffer B.

#### 4.5.2. Optimization of mRNA–pDNA Separation in pH and NaCl Gradients

A CIMmultus Swiper 1 mL chromatographic column was used by following the general handling procedures from Section 4.5.1, with the flow rate adjusted to 2 mL/min (2 CV/min). In these experiments, a sample containing 10 µg of eGFP mRNA and 10 µg pUCBS4.7 plasmid was separated. An elution gradient was performed over 20 CV, while buffer composition was varied during the evaluation as described below.

The following gradients were used:

Gradient 7. Buffer A: 10 mM MES, 10 mM HEPES, 50 mM NaCl, pH 6.0. Buffer B: 10 mM MES, 10 mM HEPES, 50 mM NaCl, pH 7.5. CIMmultus Swiper 1 mL column (2 µm), linear gradient from 100% buffer A to 100% buffer B over 20 CV at a flowrate of 2 mL/min.

Gradient 8. Buffer A: 20 mM Na-citrate, 20 mM Na-phosphate, pH 6.0. Buffer B: 20 mM Na-citrate, 20 mM Na-phosphate, pH 7.5. CIMmultus Swiper 1 mL column (2 µm), linear gradient from 100% buffer A to 100% buffer B over 20 CV at a flowrate of 2 mL/min.

Gradient 9. Buffer A: 10 mM MES, 10 mM HEPES, 150 mM NaCl, pH 6.0. Buffer B: 10 mM MES, 10 mM HEPES, 150 mM NaCl, pH 7.5. CIMmultus Swiper 1 mL column (2 µm), linear gradient from 100% buffer A to 100% buffer B over 20 CV at a flowrate of 2 mL/min.

Gradient 10. Buffer A: 50 mM Na-citrate, pH 6.0. Buffer B: 50 mM Na-citrate, 2 M NaCl, pH 6.0. CIMmultus Swiper 1 mL column (2 µm), linear gradient from 100% buffer A to 100% buffer B over 20 CV at a flowrate of 2 mL/min.

Gradient 11. Buffer A: 50 mM Na-citrate, pH 5.0. Buffer B: 50 mM Na-citrate, 2 M NaCl, pH 5.0. CIMmultus Swiper 1 mL column (2 µm), linear gradient from 100% buffer A to 100% buffer B over 20 CV at a flowrate of 2 mL/min.

The resolution between mRNA and pDNA (*R*) was calculated according to the European Pharmacopoeia, using Equation (1).

#### 4.5.3. Separation of ssRNAs of Different Sizes

ssRNA and its corresponding linear DNA template were separated for three pairs of constructs: namely, eGFP mRNA (995 nt ssRNA size, 3.3 kbp DNA size), mFIX mRNA (4000 nt ssRNA size, 6.6 kbp DNA size) and saRNA (10,000 nt ssRNA size, 12 kbp DNA size). The loading sample was composed of 10 µg of ssRNA and 5 µg of linear template DNA. A CIMmultus Swiper 1 mL chromatographic column was used for separation in Gradient 8.

The resolution between ssRNA and DNA (*R*) was calculated according to the European Pharmacopoeia, using Equation (1). Additionally, the elution recovery of each ssRNA was estimated by injecting a sample containing only ssRNA on the column or without it in triplicate and eluting it in Gradient 8. Areas of chromatographic peaks were obtained with the column (*A*_column_) or without the column (*A*_without column_) and ssRNA recovery was calculated using Equation (3).

(3) $$\;\;\;\;\;\;\;ssRNA\;recovery=100\%\times \frac{A_{column}}{A_{without\;column}}$$

#### 4.5.4. Purification of mRNA from IVT Reaction Mixture

The purification of mRNA was performed with a CIMmultus Swiper 1 mL monolithic column (Sartorius BIA Separations) with an ÄKTA pure 150M system (Cytiva) at a flowrate of 2 mL/min (2 CV/min). Prior to purification, the column was prepared as described in Section 4.5.1 and finally equilibrated with 20 CV of buffer A (20 mM citrate, 20 mM phosphate, pH 5.0). An EDTA-inactivated IVT sample containing 1.5 mg eGFP mRNA was diluted 10 times with buffer A and applied to the equilibrated column via a 2 mL capillary loop. The flow-through (FT) fraction was collected during sample application. Unbound components were washed away with 5 CV of buffer A, followed by a 10 CV wash with a high-salt buffer (20 mM citrate, 20 mM phosphate, 1 M NaCl, pH 5.0) where the wash fraction (W) was collected. Next, the column was re-equilibrated with 5 CV of buffer A, and the bound mRNA was finally eluted with linear pH gradient from 100% buffer A to 100% buffer B (20 mM citrate, 20 mM phosphate, pH 7.5) over 20 CV, where the elution fraction (EL) was collected. Column CIP was performed with 5 CV of 0.1 M NaOH.

To obtain a larger amount of mRNA for a stability study and to probe the possibility of scaling up the purification, eGFP mRNA (size 995 nt) was purified with a CIMmultus Swiper 800 mL monolithic column (Sartorius BIA Separations d.o.o.) with a simplified preparative method, following the general handling procedures from Section 4.5.1. A preparative run was performed with an ÄKTA pure 150M system (Cytiva) at its maximum flowrate of 150 mL/min (0.19 CV/min). Buffer A was 50 mM citric acid, pH 5.0, high-salt wash buffer was 50 mM citric acid, 1 M NaCl, pH 5.0 and buffer B was 100 mM Na-phosphate, pH 7.5. An 800 mL CIMmultus Swiper column was loaded with an IVT mixture containing 664 mg of eGFP mRNA, and 547 mg of eGFP mRNA (83% of the loaded amount) was successfully recovered from the column in a pH elution step with buffer B.

### 4.6. Nucleic Acids Analytics and Mass Balance Calculation

The collected fractions were analyzed using a CIMac PrimaS-0.1 Analytical Column (2 µm) to quantify mRNA and evaluate NTP and DNA template clearance, as described previously [5]. The samples were also analyzed on 1% agarose gel to obtain the nucleic acid profile of the fractions, following a previously described procedure [5]. TriTrack DNA Loading Dye (Thermo Scientific) was used for AGE and 80 ng of each sample was loaded to the gel. A GeneRuler 1 kb Plus DNA Ladder (Thermo Scientific) and RiboRuler High Range RNA Ladder (Thermo Scientific) were used as markers.

The elution recovery of mRNA in the preparative run was determined as the ratio between the amount of mRNA recovered in the elution fraction and the total amount of loaded mRNA, based on PrimaS analytics.

### 4.7. mRNA Stability Study

For the stability study, an mRNA eluate from the CIMmultus Swiper 800 mL column was buffer-exchanged (30 kDa Amicon MWCO filter, Merck) into purified H_2_O and the mRNA was further diluted to a final concentration of 500 µg/mL. The buffer-exchanged and diluted sample was split into 100 µL aliquots and incubated at 37 °C, room temperature, 4 °C, −20 °C, and −80 °C for up to 34 days. At indicated time points, an aliquot was thawed at room temperature and analyzed by AGE.

## 5. Conclusions

A new weak anion-exchanging chromatographic monolith, CIM Swiper, was characterized for the purification of model mRNA molecules. CIM Swiper’s isoelectric point was determined to be 5.3, resulting in the loss of its positive charge in a slightly acidic to neutral pH range. This enabled the binding of nucleic acids at slightly acidic pH, when the chromatographic surface was positively charged, and eluting them in ascending pH or salt gradient approaches, both conducted at room temperature. mRNA was confirmed in elution fractions in the pH range of 5–7, which is the optimal range regarding the mRNA stability. In addition, the multimodal interactions of CIM Swiper with nucleic acids enabled the separation of mRNA and contaminating DNA (pDNA). We identified NaCl, Na-phosphate, Na-citrate, and Na-EDTA as additives in the 50 mM concentration range, improving the resolution between mRNA and pDNA in combination with ascending pH gradients. When the elution of nucleic acids was performed by an ascending NaCl gradient, decreasing the working pH resulted in stronger retention and, consequently, increased the NaCl concentration required for nucleic acid elution. The acquired knowledge and understanding of this elution mechanism enabled us to develop a simple approach for the purification of model mRNA from IVT reaction by loading it in slightly acidic pH, followed by 1 M NaCl wash at the same pH. Salt wash removed IVT-related impurities from the target mRNA, especially pDNA, and was later followed by a pH elution of mRNA at around pH 6.5. All steps were performed at room temperature and the mRNA elution recovery was calculated to be 93% when purifying 1.5 mg of mRNA on a 1 mL column. The process scalability was confirmed by purifying 664 mg of mRNA on an 800 mL column with an 83% elution recovery. The obtained mRNA was proven stable for at least 34 days in purified H_2_O when stored at room temperature. Additionally, efficient elution and separation from DNA template was demonstrated for ssRNAs up to 10,000 nt in size.

The working pH range and temperature of the developed procedures are ideally suited for the enhanced stability and integrity of mRNA during purification and could therefore promote safer and better nucleic acid therapeutics. Follow-up investigations will pursue the further optimization of the capture and purification conditions for mRNA as well as the platform application of CIM Swiper for the purification of various nucleic acid modalities. Additionally, the mechanisms by which various anions affect the separation and elution of nucleic acids will be studied.

## Figures and Tables

**Table 1:** Buffer conductivity, elution pH of mRNA, difference in elution pH between mRNA and pDNA (ΔpH_mRNA-pDNA_) and resolution factor for mRNA–pDNA separation (*R*_mRNA-pDNA_) obtained using 0.1 mL CIMmic Swiper disk in Gradients 2–6.

Gradient	Additive	Conductivity (mS/cm)	Elution pH of mRNA	ΔpH_mRNA-pDNA_	*R*_mRNA-pDNA_
Gradient 2	/	1	7.03	0.14	0.4
Gradient 3	NaCl	6	6.74	0.24	2.3
Gradient 4	Na-phosphate	8	6.37	0.29	3.1
Gradient 5	Na-citrate	11	6.56	0.37	5.9
Gradient 6	Na-EDTA	11	6.65	0.29	5.8

**Table 2:** Width at half height (*w*_0.5_) for peaks corresponding to mRNA and pDNA, and resolution for mRNA–pDNA separation obtained using CIMmultus Swiper 1 mL column in Gradients 7–11.

Gradient	Elution Type	*w*_0.5 pDNA_ (min)	*w*_0.5 mRNA_ (min)	*R*_mRNA-pDNA_
Gradient 7	pH	0.32	0.50	1.8
Gradient 8	pH	0.30	0.41	2.8
Gradient 9	pH	0.31	0.47	2.7
Gradient 10	salt	0.17	0.62	3.8
Gradient 11	salt	0.17	0.83	3.4

**Table 3:** ssRNA size, template size, resolution for separation of ssRNA from DNA template, and ssRNA recovery obtained using CIMmultus Swiper 1 mL column in Gradient 8. Standard deviation for ssRNA recovery was <1% (*n* = 3).

Sample	ssRNA Size (nt)	DNA Size (kbp)	*R*_ssRNA-DNA_	*ssRNA Recovery* (%)
eGFP mRNA	995 nt	3.3	2.5	95
mFIX mRNA	4000 nt	6.6	2.8	91
saRNA	10,000 nt	12.0	2.8	80

## Data Availability

The data that support the findings of this study are available from the corresponding author upon reasonable request.

## Supplementary Materials

The following supporting information can be downloaded at: https://www.mdpi.com/article/10.3390/ijms241814267/s1.

## Abbreviations

AGE	agarose gel electrophoresis
BTP	Bis-TRIS propane
CAPS	3-cyclohexyl-3-aminopropanesulfonic acid
CIM	convective interaction media
CIP	cleaning-in-place
CV	column volume
dsDNA	double-stranded DNA
E	elution
EDTA	ethylenediaminetetraacetic acid
EtOH	ethanol
FT	flow-through
HEPES	4-(2-hydroxyethyl)piperazine-1-ethanesulfonic acid
IEP	isoelectric point
IVT	in vitro transcription
mRNA	messenger ribonucleic acid
nt	nucleotides
NTP	nucleoside triphosphate
Oligo dT	oligo deoxythymidylic acid
pKa	acid dissociation constant
polyA	polyadenylic acid
pDNA	plasmid DNA
saRNA	self-amplifying RNA
ssRNA	single-stranded RNA
W	wash
