# Supplementary material for: High Recovery Chromatographic Purification of mRNA at Room Temperature and Neutral pH
Source: Int J Mol Sci. 2023 Sep 19;24(18):14267. doi: 10.3390/ijms241814267 (PMC10532270; doi:10.3390/ijms241814267)
Supplement: Supplementary file 1 [file ijms-24-14267-s001.zip › ijms-2566156-supplementary.pdf]

Supplementary Materials

High recovery chromatographic purification of mRNA at room temperature and neutral pH

Rok Miklavčič [1, 2], Polona Megušar [1], Špela Meta Kodermac [1], Blaž Bakalar [1], Darko Dolenc [1], Rok Sekirnik [1], Aleš Štrancar [1] and Urh Černigoj [1,*]

[1] Sartorius BIA Separations d.o.o., Mirce 21, 5270 Ajdovščina, Slovenia
[2] Faculty of Medicine, University of Ljubljana, Vrazov trg 2, 1000 Ljubljana, Slovenia
* Correspondence: urh.cernigoj@biaseparations.com

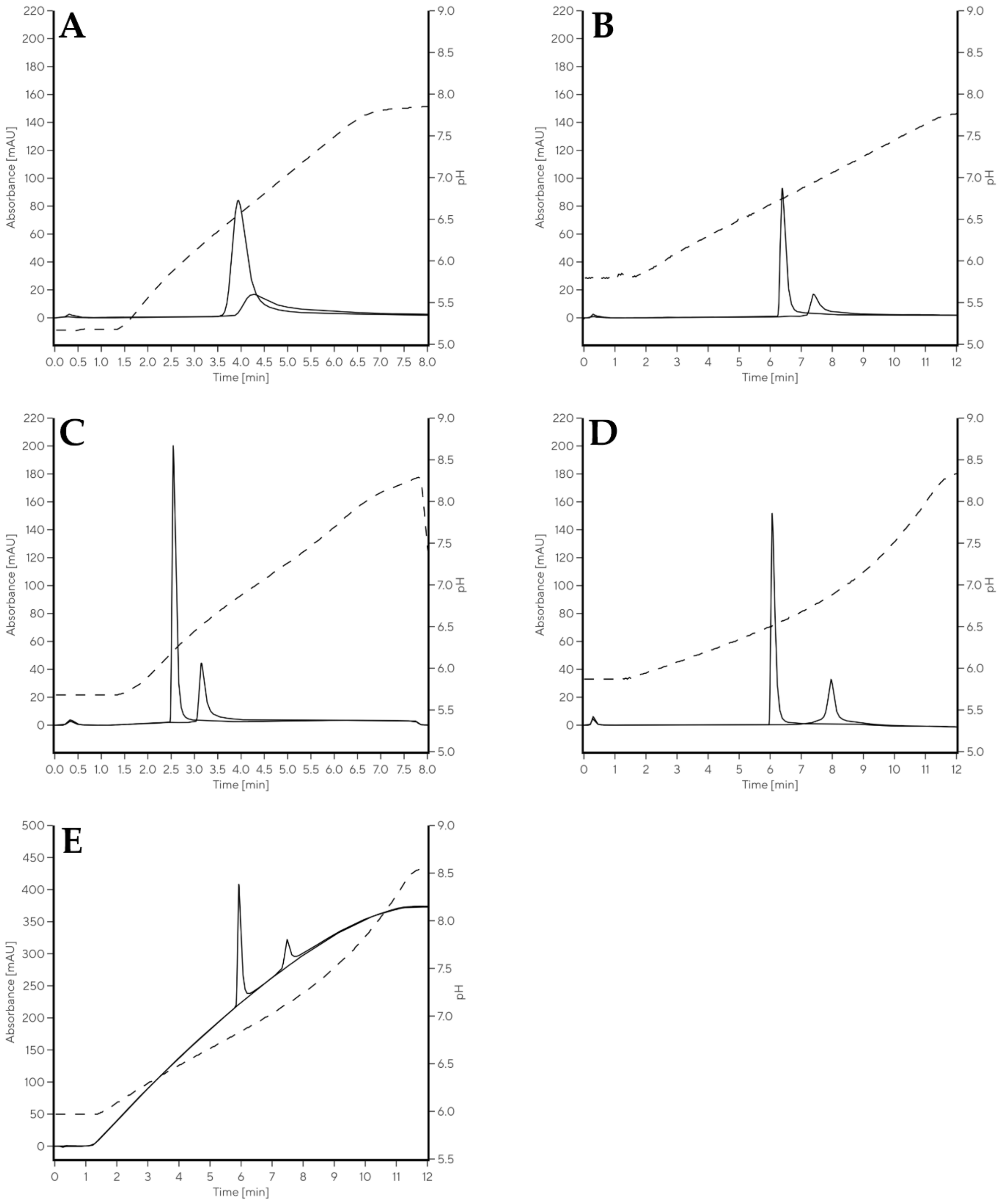

Figure S1. Chromatograms demonstrating the separation of 1 μg of eGFP mRNA and 2 μg of pUCBS4.7 plasmid in ascending pH gradient using 0.1 mL CIMmic Swiper disk in Gradients 2-6. Initial experiment was performed with no additives to pH gradient buffers (A, Gradient 2), while a 50 mM concentration of the following salts was added to pH gradient buffers during optimization of separation: NaCl (B, Gradient 3), Na-phosphate (C, Gradient 4), Na-citrate (D, Gradient 5) and Na-EDTA (E, Gradient 6). The addition of all salts improved the separation of mRNA and pDNA, but the effect of Na-phosphate, Na-citrate and Na-EDTA was more significant than that of NaCl.

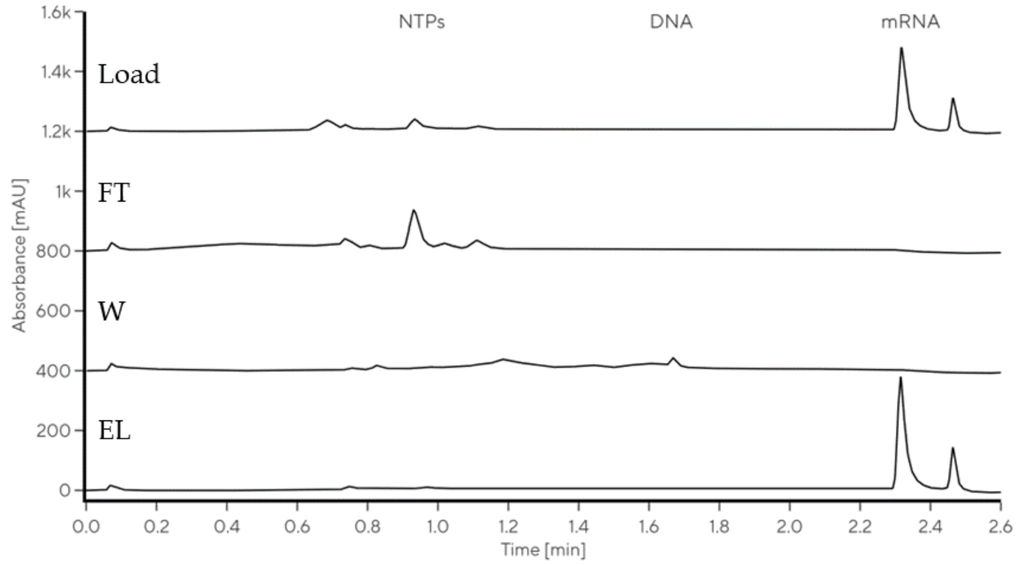

Figure S2. CIMac PrimaS analytics of fractions obtained during purification of 1.5 mg of eGFP mRNA from IVT reaction mixture using CIMmultus Swiper 1 mL monolithic column. FT: flow-through fraction, W: salt wash fraction, E: elution fraction, CIP: CIP fraction. Elution times of peaks corresponding to nucleoside triphosphates (NTPs), DNA and mRNA are annotated. Results show, that NTPs did not bind to the column under sample application conditions (FT), while contaminating DNA impurities bound to the column and were efficiently eluted with a salt wash (W). The eluted mRNA was free from detectable process-related impurities with such a method (EL).
